# Sociodemographic variation in the use of chemotherapy and radiotherapy in patients with stage IV lung, oesophageal, stomach and pancreatic cancer: evidence from population-based data in England during 2013–2014

**DOI:** 10.1038/s41416-018-0028-7

**Published:** 2018-05-10

**Authors:** Katherine E Henson, Anna Fry, Georgios Lyratzopoulos, Michael Peake, Keith J Roberts, Sean McPhail

**Affiliations:** 1 National Cancer Registration and Analysis Service, Public Health England, Skipton House, London, SE1 6LH UK; 2 Cancer Research UK, Angel Building, London, EC1V 4AD UK; 3 ECHO (Epidemiology of Cancer Healthcare and Outcomes) Group, Department of Behavioural Science and Health, University College London, Gower Street, Bloomsbury, London, WC1E 6BT UK; 4 University of Leicester, Glenfield Hospital, Groby Road, Leicester, LE3 9QP UK; 5 Centre for Cancer Outcomes, University College London Hospitals (UCLH) Cancer Collaborative, UCLH Cancer Division, 47 Wimpole Street, London, W1G 8SE UK; 6 Nuffield House, University Hospitals Birmingham, Edgbaston, Birmingham, B15 2TH UK

**Keywords:** Cancer therapy, Palliative care, Cancer epidemiology

## Abstract

**Background:**

Sociodemographic inequalities in cancer treatment have been generally described, but there is little evidence regarding patients with advanced cancer. Understanding variation in the management of these patients may provide insights into likely mechanisms leading to inequalities in survival.

**Methods:**

We identified 50,232 patients with stage IV lung, oesophageal, pancreatic and stomach cancer from the English national cancer registry. A generalised linear model with a Poisson error structure was used to explore variation in radiotherapy and chemotherapy within 6 months from diagnosis by age, sex, deprivation, ethnicity, cancer site, comorbidity and, additionally, performance status.

**Results:**

There was substantial variation by cancer site, large gradients by age, and non-trivial associations with comorbidity and deprivation. After full adjustment, more deprived patients were consistently least likely to be treated with chemotherapy alone or chemotherapy and radiotherapy combined compared with less deprived patients with equally advanced disease stage (treatment rate ratio: 0.82 95% CI (0.78, 0.87) for CT, 0.78 95% CI (0.71, 0.85) for CTRT *p* < 0.0001).

**Conclusions:**

There was marked variation in the management of patients with stage IV cancer. Routinely collected data could be used for surveillance across all cancers to help reduce treatment variation and optimise outcomes among patients with advanced cancer.

## Introduction

While much previous evidence documents sociodemographic variation in cancer treatment,^1–7^ particularly surgery with curative intent^8–11^ and lung cancer,^12,13^ most studies thus far do not take into account potential confounding by stage at diagnosis. Relatively little attention has been paid to the management of patients with stage IV disease, particularly using population-based data. While for most such patients treatment will be administered with non-curative intent, variation in management can translate to substantial differences in survival. These considerations are particularly applicable to cancer sites with large proportions of patients diagnosed with stage IV disease.

In England, routine monitoring of treatment of cancer has historically not been available, particularly for radiotherapy (RT) and chemotherapy (CT). This has historically been due to limited data availability and completeness. National audit reports routinely published treatment rates for some specific cancer sites and stages; however limited statistical adjustments for sociodemographic and clinical factors were included.^14–16^

Treatment among patients with advanced cancers is influenced by numerous factors such as performance status (a measure of general well-being, and the ability of a cancer patient to maintain their normal daily activities), patient age, presence and severity of comorbidity, perceived benefits and side-effects of treatment and patient choice. These are largely understandable clinically as issues that influence a patient’s likely tolerance to treatment, like age, though probably to a lesser extent.^1,7,17,18^ However, recent studies have discussed the sometimes problematic assumed relationship between frailty and age, and that more sophisticated measures of frailty should be employed during treatment decisions to reduce inequalities and offer optimum treatment.^19^ England has an increasingly robust system of data collection among patients with cancer and thus it is now possible to study variation in treatment whilst adjusting for these important factors.

Motivated by the above considerations, we aimed to describe the population level uptake and variation of treatment among patients with stage IV lung, pancreas, stomach and oesophageal cancer, with a particular hypothesis that sociodemographic factors explain some of the variation. Understanding variation in the management of these patients may provide insights into likely mechanisms leading to inequalities in survival and help develop interventions to reduce them. We focused on four cancer sites with a high proportion of stage IV cancers diagnosed, and where prior evidence indicates that use of surgery in patients with metastatic disease is rare (this was supported in our cohort of stage IV patients, with less than 5% recorded to have received surgery intended to remove the primary tumour). Thus, CT and RT are the main treatment modalities for stage IV disease, and intent was assumed to be almost universally non-curative.

## Patients and Methods

### Study population

Patients diagnosed with lung (ICD-10 C34, divided into small cell (SCLC) (Supplementary Table 1) and non-small cell (NSCLC), selected as lung cases not identified as small cell), oesophagus (C15), pancreas (C25) and stomach (C16) cancer during 2013–2014 were identified from Public Health England’s national cancer registration data. All patients were resident in England and aged 15–99 years at diagnosis. Patients identified through their death certificate only (18 patients) were excluded, as were patients with mis-ordered dates (13 patients), for example a date of death prior to diagnosis.

Cancer registration data, which in isolation contains some treatment data, was linked with the Radiotherapy Dataset (RTDS)^20^ and Systemic Anti-Cancer Therapy (SACT)^21^ data to ascertain treatment uptake for each patient from 30 days prior to and 183 days following the date of cancer diagnosis. The datasets were linked on NHS number; therefore the additional treatment-specific datasets (RTDS and SACT) were only used for patients who were diagnosed with one tumour in any 6 month period. The outcome variables were therefore RT (without CT), CT (without RT) and CTRT, which may have been delivered independently of each other. Immunotherapies are captured in SACT, and are thus included as ‘CT’. Brachytherapy episodes were excluded, as were SACT records for hormonal therapy and treatment other than active anti-cancer therapy (such drug therapies for the management of bone metastasis such as zoledronic acid, pamidronate and denosumab).

To calculate the income deprivation quintile, each patient’s postcode at diagnosis (at the Lower Super Output Area geography) was linked to the income domain of the Index of Multiple Deprivation 2010.^22^ Equal population quintiles were derived over the whole of England from the income domain score, and each cancer patient was assigned the income deprivation quintile of their local geography. Ethnicity was self-reported and recorded in the hospital patient administration systems. If the patient’s ethnicity was unknown in the cancer registration data, the modal value from the Hospital Episode Statistics (HES) data^23^ was used. Ethnicity is recorded for each hospital episode, and the most common ethnicity was chosen (or the most recent of these in the event of a tied number of records). The comorbidity index was derived as per the Charlson comorbidity index lookup table^24^ using in-patient HES data, with the same methodology as described by Maringe et al (2017),^25^ but with a different time window: from 27 months to 3 months prior to the cancer diagnosis. Performance status was obtained from both cancer registration and SACT data, and is as recorded by the multi-disciplinary team (MDT). The performance status was measured at both diagnosis and at the start of each chemotherapy regimen, thus if a patient was assigned two performance status values, the maximum (most disadvantageous) value was chosen. Vital status was ascertained from the Office of National Statistics (ONS) mortality data.

### Statistical analysis

Each individual entered the analysis at 31 days before the date of cancer diagnosis (to allow for treatment commencing prior to diagnosis date, as defined under the European Network of Cancer Registries (ENCR) rules which prioritise date of pathology^26^) and was censored at the earliest of date of first treatment, death (from the patient’s vital status), or end of the 6 months post-diagnosis follow up. 1.0% of RT events and 0.2% of CT events occurred in the 31 day period prior to ENCR cancer diagnosis date.

To account for varied survival times among the cohort, a model was chosen to account for time at risk, as defined above. Hazard rate ratios (for treatment vs. no treatment, henceforth called treatment rate ratios (TRR)) were calculated using a generalised linear model with a Poisson error structure on collapsed data^27^ where the treatment rate in the reference population was taken as zero. This was performed using the STRS program in Stata 13.1.^27,28^ This modelling approach for excess mortality is the analogue of the relative survival approach. Individual patient-level data was used; therefore exact survival times were available. The data was collapsed for ease of computation, as Dickman and colleagues demonstrated equivalent results to un-collapsed data.^27^ The excess hazards are assumed to be constant throughout each month of follow-up therefore a Poisson process is assumed for the rate of first treatments per follow-up interval.^27^ The hazard is dependent on a subset of the explanatory variables (age, sex and follow-up period). Where patients received both RT and CT, the start date of this combined treatment was considered to be the first event.

Separate regression models were performed for: RT (without CT), CT (without RT) and CTRT. The factors investigated were: cancer site, sex, age (<50, 50–59, 60–69, 70–79, 80+), sociodemographic deprivation (income deprivation quintile: 1—least deprived, 2, 3, 4, 5—most deprived), ethnicity (white, non-white, unknown) and Charlson comorbidity index (0, 1, 2, 3+).^29^

Supplementary analyses included performance status as an explanatory variable in the multivariable regression model (missing data precluded its use as a main explanatory variable). Due to theoretical concerns of associations of treatment modality with morphology subtypes, we also conducted additional analysis of dominant subtypes restricting the tumour cohort to oesophageal, pancreatic and stomach adenocarcinomas (ICD-O-2 morphologies 8140-8576).

Tests for heterogeneity were performed jointly across all categories of each variable, using a post-estimation Wald test. Statistical significance was defined as two-sided with a *p* < 0.05.

## Results

### Patient characteristics

50,232 patients with stage IV lung, oesophageal, stomach and pancreatic cancers were diagnosed during 2013–2014, and cohort characteristics for each of the cancer sites are summarised in Table 1. Across all sites, 24% received CT (without RT), 13% of patients received RT (without CT) and 11% received both CT and RT (CTRT), which varied by cancer type from 1% for CTRT and RT among patients with pancreatic cancer to 37% for CT among patients with oesophageal cancer (Table 2). The proportion of patients who died during the 6 month follow-up period varied from 57% to 78% for oesophageal and pancreatic cancer, respectively, and among these patients the median survival time varied from 41 days (pancreatic and SCLC) to 63 days (oesophageal) (Supplementary Table 2).

**Table 1:** Patient characteristics of stage IV lung, oesophageal, pancreatic and stomach cancers diagnosed in 2013–2014 in England

	Number of cancers diagnosed										Total
	NSCLC	%	SCLC	%	Oesophagus	%	Stomach	%	Pancreas	%	
Total	30,431		5,079		4,023		3,588		7,111		50,232
*Year of diagnosis*											
2013	14,979	49	2,532	50	1,847	46	1,783	50	3,288	46	24,429
2014	15,452	51	2,547	50	2,176	54	1,805	50	3,823	54	25,803
*Sex*											
Male	16,839	55	2,712	53	2,898	72	2,401	67	3,715	52	28,565
Female	13,592	45	2,367	47	1,125	28	1,187	33	3,396	48	21,667
*Age at cancer diagnosis*											
<50	847	3	148	3	175	4	262	7	315	4	1,747
50–59	2,953	10	697	14	557	14	366	10	829	12	5,402
60–69	8,135	27	1,761	35	1,218	30	770	21	1,881	26	13,765
70–79	10,075	33	1,771	35	1,234	31	1,180	33	2,275	32	16,535
80+	8,421	28	702	14	839	21	1,010	28	1,811	25	12,783
*Charlson comorbidity index*											
0	22,840	75	3,922	77	3,285	82	2,816	78	5,461	77	38,324
1	3,661	12	578	11	373	9	395	11	845	12	5,852
2	2,007	7	331	7	192	5	228	6	435	6	3,193
3+	1,923	6	248	5	173	4	149	4	370	5	2,863
*Deprivation quintile*											
1: least deprived	4,377	14	623	12	731	18	601	17	1,445	20	7,777
2	5,516	18	887	17	832	21	733	20	1,531	22	9,499
3	6,411	21	1,009	20	857	21	741	21	1,577	22	10,595
4	6,862	23	1,192	23	832	21	769	21	1,349	19	11,004
5: most deprived	7,265	24	1,368	27	771	19	744	21	1,209	17	11,357
*Ethnicity*											
White	28,133	92	4,817	95	3,781	94	3,198	89	6,411	90	46,340
Non-white	1,182	4	120	2	91	2	264	7	376	5	2,033
Unknown	1,116	4	142	3	151	4	126	4	324	5	1,859
*Performance status*											
0	2,567	8	414	8	586	15	369	10	418	6	4,354
1+	14,985	49	3,109	61	1,342	33	1,164	32	1,161	16	21,761
Unknown	12,879	42	1,556	31	2,095	52	2,055	57	5,532	78	24,117

*NSCLC* non-small cell lung cancer, *SCLC* small cell lung cancer

**Table 2:** Treatment rate ratios for chemotherapy alone (CT), radiotherapy alone (RT) and chemotherapy & radiotherapy (CTRT) within 6 months of diagnosis of lung, oesophageal, pancreatic and stomach cancers in 2013–2014 from three unadjusted and three fully adjusted multivariable models with cancer site, sex, age at diagnosis, deprivation, ethnicity and Charlson comorbidity index

	RT			CT			CTRT		
	%^a^	TRR (95% CI)		%^a^	TRR (95% CI)		%^a^	TRR (95% CI)	
		Unadjusted	Fully adjusted		Unadjusted	Fully adjusted		Unadjusted	Fully adjusted
*Sex*									
Male	13	Ref	Ref	24	Ref	Ref	11	Ref	Ref
Female	12	0.88 (0.84, 0.92)	0.85 (0.81, 0.89)	23	0.90 (0.86, 0.93)	0.97 (0.93, 1.00)	12	1.02 (0.97, 1.08)	0.95 (0.91, 1.01)
*p* for heterogeneity^b^		<0.0001	<0.0001		<0.0001	0.08		0.36	0.08
*Age at cancer diagnosis*									
<50	9	0.57 (0.48, 0.67)	0.65 (0.55, 0.77)	45	1.35 (1.25, 1.46)	1.24 (1.15, 1.34)	21	1.11 (0.99, 1.24)	1.41 (1.26, 1.58)
50–59	12	0.86 (0.79, 0.94)	0.91 (0.83, 0.99)	34	1.04 (0.99, 1.10)	1.01 (0.95, 1.06)	22	1.27 (1.18, 1.36)	1.33 (1.24, 1.43)
60–69	13	Ref	Ref	32	Ref	Ref	17	Ref	Ref
70–79	14	1.21 (1.14, 1.29)	1.20 (1.13, 1.28)	24	0.78 (0.75, 0.82)	0.79 (0.76, 0.83)	9	0.55 (0.51, 0.58)	0.56 (0.53, 0.60)
80+	12	1.22 (1.14, 1.30)	1.13 (1.05, 1.21)	7	0.21 (0.20, 0.23)	0.22 (0.21, 0.24)	2	0.14 (0.12, 0.15)	0.15 (0.13, 0.17)
*p* for heterogeneity^b^		<0.0001	<0.0001		<0.0001	<0.0001		<0.0001	<0.0001
*Charlson comorbidity index*									
0	13	Ref	Ref	26	Ref	Ref	13	Ref	Ref
1	12	1.01 (0.93, 1.09)	0.95 (0.88, 1.03)	19	0.76 (0.71, 0.81)	0.86 (0.81, 0.91)	9	0.70 (0.63, 0.76)	0.81 (0.74, 0.89)
2	12	1.08 (0.98, 1.20)	0.99 (0.89, 1.09)	16	0.63 (0.58, 0.69)	0.74 (0.68, 0.81)	8	0.64 (0.56, 0.73)	0.78 (0.69, 0.89)
3+	12	1.15 (1.03, 1.28)	0.98 (0.88, 1.10)	11	0.43 (0.39, 0.48)	0.57 (0.51, 0.64)	4	0.32 (0.26, 0.38)	0.41 (0.34, 0.50)
*p* for heterogeneity^b^		0.04	0.67		<0.0001	<0.0001		<0.0001	<0.0001
*Deprivation quintile*									
1: least deprived	12	Ref	Ref	27	Ref	Ref	12	Ref	Ref
2	12	1.04 (0.96, 1.13)	1.02 (0.94, 1.11)	25	0.95 (0.90, 1.01)	0.95 (0.90, 1.01)	12	1.04 (0.95, 1.14)	0.96 (0.88, 1.04)
3	13	1.12 (1.03, 1.22)	1.09 (1.00, 1.18)	23	0.88 (0.83, 0.93)	0.89 (0.84, 0.94)	11	1.01 (0.93, 1.10)	0.90 (0.82, 0.98)
4	13	1.09 (1.00, 1.18)	1.04 (0.96, 1.13)	23	0.86 (0.81, 0.92)	0.86 (0.81, 0.91)	11	1.04 (0.96, 1.14)	0.84 (0.77, 0.91)
5: most deprived	13	1.14 (1.05, 1.23)	1.08 (1.00, 1.18)	22	0.85 (0.80, 0.90)	0.82 (0.78, 0.87)	11	1.07 (0.98, 1.17)	0.78 (0.71, 0.85)
*p* for heterogeneity^b^		0.01	0.18		<0.0001	<0.0001		0.48	<0.0001
*Ethnicity*									
White	13	Ref	Ref	24	Ref	Ref	11	Ref	Ref
Non-white	12	0.85 (0.75, 0.96)	0.91 (0.80, 1.04)	29	1.13 (1.04, 1.22)	1.01 (0.92, 1.10)	14	1.07 (0.95, 1.21)	1.07 (0.95, 1.21)
Unknown	11	0.93 (0.81, 1.08)	0.93 (0.81, 1.07)	17	0.80 (0.71, 0.89)	0.75 (0.67, 0.84)	7	0.67 (0.56, 0.79)	0.67 (0.56, 0.79)
*p* for heterogeneity^b^		0.03	0.24		<0.0001	<0.0001		<0.0001	<0.0001
*Cancer site*									
NSCLC	18	Ref	Ref	18	Ref	Ref	12	Ref	Ref
SCLC	5	0.25 (0.22, 0.29)	0.26 (0.23, 0.29)	33	1.89 (1.79, 1.99)	1.61 (1.52, 1.70)	32	3.31 (3.12, 3.51)	2.91 (2.74, 3.09)
Oesophagus	10	0.47 (0.43, 0.52)	0.47 (0.42, 0.52)	37	2.04 (1.92, 2.16)	1.88 (1.77, 1.99)	8	0.57 (0.51, 0.64)	0.47 (0.42, 0.53)
Stomach	5	0.25 (0.21, 0.29)	0.25 (0.21, 0.29)	36	2.14 (2.02, 2.28)	2.16 (2.03, 2.30)	4	0.26 (0.21, 0.31)	0.23 (0.19, 0.27)
Pancreas	1	0.08 (0.07, 0.10)	0.09 (0.07, 0.10)	27	1.86 (1.76, 1.96)	1.78 (1.68, 1.87)	1	0.12 (0.10, 0.15)	0.10 (0.09, 0.13)
*p* for heterogeneity^b^		<0.0001	<0.0001		<0.0001	<0.0001		<0.0001	<0.0001

CI confidence interval, *NSCLC* non-small cell lung cancer, *SCLC* small cell lung cancer, *TRR* treatment rate ratio.

^a^ Proportion of patients recorded to receive treatment in each category.

^b^ Performed jointly across all categories of each variable, using a post-estimation Wald test

### Overall variation in treatment usage

Treatment rates varied significantly across cancer sites (*p* < 0.0001). RT was used more frequently in patients with NSCLC, CT in patients with stomach cancer and CTRT in patients with SCLC (Table 2). Full adjustment attenuated crude TRR values up to 33% (Table 2), therefore, only adjusted results are presented in Table 3 onwards. Across all cancer sites, there was a significant association between female sex and lower rate of RT (*p* < 0.0001), but there was no evidence for association by sex for CT or CTRT use (*p* = 0.08 for both). The sex gradient in RT use among NSCLC was driven by non-squamous tumours (Supplementary Table 3). There was no difference in treatment use by known ethnicity. Analyses of radiotherapy and chemotherapy independently of the other treatment found no substantive differences, except for an association between increasing age at diagnosis and decreasing use of radiotherapy (Supplementary Table 4). Restriction to upper gastrointestinal adenocarcinomas (Supplementary Table 5) demonstrated highly concordant patterns of variation with that observed for results of all morphologies combined for upper gastrointestinal cancers (Table 2), with an expected loss of significance due to diminished sample size among stratified results (data not shown).

**Table 3:** Treatment rate ratios for radiotherapy alone (RT) within 6 months of diagnosis of lung, oesophageal, pancreatic and stomach cancers in 2013–2014, from a fully adjusted multivariable model with sex, age at diagnosis deprivation, ethnicity and Charlson comorbidity index, stratified by cancer site

	NSCLC		SCLC		Oesophagus		Stomach		Pancreas	
	%^a^	TRR (95% CI)	%^a^	TRR (95% CI)	%^a^	TRR (95% CI)	%^a^	TRR (95% CI)	%^a^	TRR (95% CI)
*Sex*										
Male	19	Ref	6	Ref	9	Ref	5	Ref	1	Ref
Female	16	0.82 (0.77, 0.86)	5	0.85 (0.67, 1.09)	14	1.41 (1.15, 1.74)	5	0.87 (0.63, 1.19)	1	1.03 (0.69, 1.52)
*p* for heterogeneity^b^		<0.0001		0.19		0.001		0.39		0.89
*Age at cancer diagnosis*										
<50	15	0.69 (0.57, 0.83)	1	0.35 (0.09, 1.43)	7	0.63 (0.34, 1.18)	1	0.31 (0.09, 1.03)	1	0.65 (0.23, 1.86)
50–59	19	0.93 (0.84, 1.03)	4	1.14 (0.73, 1.77)	6	0.64 (0.43, 0.94)	4	0.99 (0.51, 1.92)	1	0.76 (0.38, 1.51)
60–69	19	Ref	4	Ref	9	Ref	4	Ref	2	Ref
70–79	19	1.13 (1.06, 1.21)	6	1.76 (1.28, 2.40)	12	1.34 (1.04, 1.72)	6	1.80 (1.15, 2.80)	2	1.03 (0.63, 1.66)
80+	15	0.96 (0.89, 1.04)	10	3.58 (2.54, 5.05)	14	1.86 (1.42, 2.44)	7	2.56 (1.64, 4.00)	1	0.82 (0.46, 1.45)
*p* for heterogeneity^b^		<0.0001		<0.0001		<0.0001		<0.0001		0.78
*Charlson comorbidity index*										
0	18	Ref	5	Ref	10	Ref	5	Ref	1	Ref
1	16	0.93 (0.85, 1.01)	5	0.95 (0.64, 1.42)	9	0.89 (0.63, 1.26)	7	1.48 (0.99, 2.22)	2	1.64 (0.97, 2.76)
2	16	0.94 (0.84, 1.05)	7	1.43 (0.93, 2.19)	13	1.14 (0.74, 1.74)	7	1.32 (0.77, 2.26)	1	0.79 (0.29, 2.16)
3+	15	0.92 (0.82, 1.04)	10	1.80 (1.17, 2.77)	10	0.97 (0.60, 1.56)	7	1.27 (0.66, 2.42)	2	1.55 (0.67, 3.59)
*p* for heterogeneity^b^		0.19		0.02		0.83		0.23		0.20
*Deprivation quintile*										
1: least deprived	18	Ref	5	Ref	11	Ref	5	Ref	2	Ref
2	18	1.01 (0.92, 1.11)	7	1.30 (0.85, 1.99)	11	1.07 (0.79, 1.45)	6	1.11 (0.71, 1.74)	1	0.72 (0.40, 1.28)
3	18	1.10 (1.01, 1.21)	5	1.02 (0.66, 1.57)	11	1.03 (0.76, 1.39)	6	1.01 (0.63, 1.60)	2	0.87 (0.51, 1.51)
4	17	1.05 (0.96, 1.15)	5	1.15 (0.75, 1.74)	10	0.96 (0.71, 1.32)	5	0.92 (0.58, 1.48)	1	0.65 (0.34, 1.22)
5: most deprived	18	1.11 (1.02, 1.21)	4	0.97 (0.63, 1.47)	9	0.87 (0.63, 1.21)	4	0.66 (0.39, 1.11)	1	0.67 (0.35, 1.29)
*p* for heterogeneity^b^		0.05		0.49		0.77		0.31		0.58
*Ethnicity*										
White	18	Ref	5	Ref	10	Ref	5	Ref	1	Ref
Non-white	18	0.86 (0.75, 1.00)	8	1.32 (0.68, 2.59)	12	1.24 (0.68, 2.28)	6	1.68 (0.99, 2.86)	2	1.19 (0.51, 2.78)
Unknown	15	0.89 (0.76, 1.04)	8	1.82 (0.99, 3.35)	5	0.52 (0.26, 1.05)	5	1.22 (0.54, 2.77)	2	1.66 (0.72, 3.82)
*p* for heterogeneity^b^		0.05		0.12		0.14		0.15		0.46

CI confidence interval, *NSCLC* non-small cell lung cancer, *SCLC* small cell lung cancer, *TRR* treatment rate ratio.

^a^ Proportion of patients recorded to receive treatment in each category.

^b^ Performed jointly across all categories of each variable, using a post-estimation Wald test

### Variation by age at cancer diagnosis

For all cancer sites (except pancreas, *p* = 0.78) there was a positive association between older age and higher RT rate (*p* < 0.0001; Table 3). The steepest association was for SCLC (TRR: 0.35 (95% CI 0.09, 1.43) for ages <50 to 3.58 (95% CI 2.54, 5.05) for ages 80+). There was very strong statistical evidence for associations between increasing age and lower CTRT use for all sites (*p* < 0.0001; Table 4), this association being steeper for pancreatic cancer, with TRRs ranging from 1.77 (95% CI 0.86, 3.63) for ages <50 to 0.28 (95% CI 0.12, 0.68) for ages 80+. Older age was associated with a lower rate of CT (*p* < 0.0001; Table 5), except for SCLC, with the highest use in patients aged 70–79 (TRR: 1.26 (95% CI 1.13, 1.41)).

**Table 4:** Treatment rate ratios for chemotherapy & radiotherapy (CTRT) within 6 months of diagnosis of lung, oesophageal, pancreatic and stomach cancers in 2013–2014, from a fully adjusted multivariable model with sex, age at diagnosis, deprivation, ethnicity and Charlson comorbidity index, stratified by cancer site

	NSCLC		SCLC		Oesophagus		Stomach		Pancreas	
	%^a^	TRR (95% CI)	%^a^	TRR (95% CI)	%^a^	TRR (95% CI)	%^a^	TRR (95% CI)	%^a^	TRR (95% CI)
*Sex*										
Male	12	Ref	31	Ref	9	Ref	4	Ref	1	Ref
Female	12	0.96 (0.90, 1.03)	33	1.00 (0.90, 1.10)	6	0.82 (0.63, 1.07)	3	0.63 (0.42, 0.95)	1	1.06 (0.71, 1.57)
*p* for heterogeneity^b^		0.22		0.93		0.15		0.03		0.79
*Age at cancer diagnosis*										
<50	29	1.52 (1.33, 1.74)	50	1.31 (1.03, 1.66)	10	0.87 (0.52, 1.44)	9	1.69 (1.00, 2.85)	3	1.77 (0.86, 3.63)
50 – 59	25	1.36 (1.25, 1.49)	47	1.26 (1.10, 1.43)	14	1.37 (1.03, 1.82)	5	1.04 (0.60, 1.78)	3	2.07 (1.24, 3.48)
60 – 69	18	Ref	39	Ref	10	Ref	5	Ref	2	Ref
70 – 79	9	0.53 (0.49, 0.58)	25	0.59 (0.53, 0.67)	7	0.75 (0.57, 0.98)	3	0.56 (0.35, 0.88)	1	0.77 (0.45, 1.32)
80+	2	0.11 (0.10, 0.13)	11	0.28 (0.22, 0.35)	2	0.24 (0.14, 0.40)	1	0.26 (0.13, 0.51)	0	0.28 (0.12, 0.68)
*p* for heterogeneity^b^		<0.0001		<0.0001		<0.0001		<0.0001		<0.0001
*Charlson comorbidity index*										
0	13	Ref	34	Ref	9	Ref	4	Ref	2	Ref
1	8	0.73 (0.65, 0.83)	28	0.88 (0.75, 1.04)	9	1.27 (0.88, 1.83)	3	0.93 (0.50, 1.74)	2	1.19 (0.66, 2.14)
2	7	0.71 (0.60, 0.84)	27	0.92 (0.74, 1.14)	5	0.76 (0.41, 1.44)	3	1.06 (0.49, 2.30)	1	0.66 (0.21, 2.11)
3+	3	0.35 (0.27, 0.45)	15	0.50 (0.36, 0.70)	4	0.71 (0.33, 1.51)	2	0.80 (0.25, 2.55)	0	0.27 (0.04, 1.92)
*p* for heterogeneity^b^		<0.0001		0.0004		0.33		0.98		0.45
*Deprivation quintile*										
1: least deprived	13	Ref	32	Ref	11	Ref	4	Ref	2	Ref
2	13	0.98 (0.88, 1.10)	32	0.91 (0.76, 1.09)	9	0.86 (0.63, 1.19)	3	0.93 (0.52, 1.66)	2	1.00 (0.56, 1.77)
3	11	0.86 (0.77, 0.96)	35	1.03 (0.87, 1.23)	7	0.70 (0.50, 0.98)	4	1.16 (0.67, 2.01)	1	0.71 (0.38, 1.33)
4	11	0.82 (0.74, 0.92)	31	0.87 (0.73, 1.03)	8	0.71 (0.51, 0.99)	4	0.96 (0.55, 1.69)	1	1.00 (0.55, 1.82)
5: most deprived	11	0.77 (0.69, 0.85)	30	0.81 (0.68, 0.96)	7	0.61 (0.43, 0.87)	3	0.90 (0.50, 1.62)	1	0.84 (0.44, 1.63)
*p* for heterogeneity^b^		<0.0001		0.009		0.04		0.91		0.79
Ethnicity										
White	12	Ref	32	Ref	8	Ref	4	Ref	1	Ref
Non-white	18	1.16 (1.01, 1.33)	31	0.83 (0.59, 1.15)	10	1.24 (0.64, 2.43)	5	1.08 (0.59, 1.97)	1	0.39 (0.12, 1.26)
Unknown	8	0.67 (0.55, 0.83)	21	0.73 (0.51, 1.05)	3	0.39 (0.16, 0.95)	2	0.53 (0.13, 2.16)	1	0.46 (0.11, 1.88)
*p* for heterogeneity^b^		0.0001		0.13		0.09		0.65		0.17

CI confidence interval, *NSCLC* non-small cell lung cancer, *SCLC* small cell lung cancer, *TRR* treatment rate ratio.

^a^ Proportion of patients recorded to receive treatment in each category.

^b^ Performed jointly across all categories of each variable, using a post-estimation Wald test

**Table 5:** Treatment rate ratios for chemotherapy alone (CT) within 6 months of diagnosis of lung, oesophageal, pancreatic and stomach cancers in 2013–2014, from a fully adjusted multivariable model with sex, age at diagnosis, deprivation, ethnicity and Charlson comorbidity index, stratified by cancer site

	NSCLC		SCLC		Oesophagus		Stomach		Pancreas	
	%^a^	TRR (95% CI)	%^a^	TRR (95% CI)	%^a^	TRR (95% CI)	%^a^	TRR (95% CI)	%^a^	TRR (95% CI)
*Sex*										
Male	18	Ref	33	Ref	40	Ref	36	Ref	28	Ref
Female	19	1.02 (0.97, 1.08)	33	0.98 (0.89, 1.08)	29	0.84 (0.74, 0.95)	34	0.98 (0.87, 1.11)	25	0.97 (0.88, 1.06)
*p* for heterogeneity^b^		0.39		0.74		0.007		0.78		0.48
*Age at cancer diagnosis*										
<50	33	1.18 (1.04, 1.34)	36	0.96 (0.72, 1.26)	61	1.32 (1.08, 1.63)	61	1.17 (0.97, 1.41)	56	1.51 (1.28, 1.78)
50–59	28	1.03 (0.95, 1.12)	30	0.79 (0.67, 0.92)	52	1.07 (0.93, 1.24)	54	1.06 (0.89, 1.26)	42	1.10 (0.97, 1.25)
60–69	26	Ref	35	Ref	48	Ref	53	Ref	38	Ref
70–79	19	0.78 (0.73, 0.83)	38	1.26 (1.13, 1.41)	35	0.73 (0.64, 0.82)	36	0.62 (0.54, 0.71)	25	0.68 (0.61, 0.76)
80+	5	0.21 (0.19, 0.23)	22	0.82 (0.69, 0.98)	8	0.16 (0.12, 0.20)	10	0.16 (0.13, 0.20)	5	0.15 (0.12, 0.19)
*p* for heterogeneity^b^		<0.0001		<0.0001		<0.0001		<0.0001		<0.0001
*Charlson comorbidity index*										
0	20	Ref	34	Ref	40	Ref	38	Ref	29	Ref
1	14	0.84 (0.77, 0.92)	33	0.97 (0.83, 1.13)	27	0.76 (0.62, 0.93)	31	0.92 (0.76, 1.12)	23	0.85 (0.73, 0.98)
2	12	0.72 (0.63, 0.82)	29	0.84 (0.69, 1.04)	22	0.64 (0.47, 0.86)	26	0.75 (0.57, 0.98)	18	0.76 (0.60, 0.96)
3+	7	0.49 (0.41, 0.58)	32	1.06 (0.84, 1.33)	14	0.43 (0.28, 0.64)	15	0.53 (0.35, 0.80)	12	0.54 (0.40, 0.73)
*p* for heterogeneity^b^		<0.0001		0.39		<0.0001		0.004		<0.0001
*Deprivation quintile*										
1: least deprived	21	Ref	32	Ref	40	Ref	38	Ref	30	Ref
2	19	0.92 (0.84, 1.01)	33	1.05 (0.87, 1.25)	35	0.85 (0.72, 1.00)	38	1.04 (0.88, 1.24)	29	0.99 (0.87, 1.13)
3	17	0.85 (0.77, 0.92)	32	0.97 (0.82, 1.16)	37	0.92 (0.79, 1.08)	36	0.98 (0.82, 1.18)	27	0.92 (0.80, 1.05)
4	17	0.81 (0.75, 0.89)	34	1.10 (0.92, 1.30)	37	0.90 (0.76, 1.05)	34	0.87 (0.72, 1.03)	24	0.82 (0.71, 0.95)
5: most deprived	17	0.77 (0.71, 0.84)	35	1.16 (0.98, 1.37)	34	0.77 (0.65, 0.91)	32	0.81 (0.68, 0.98)	24	0.78 (0.67, 0.91)
*p* for heterogeneity^b^		<0.0001		0.13		0.03		0.02		0.002
*Ethnicity*										
White	18	Ref	34	Ref	37	Ref	36	Ref	27	Ref
Non-white	25	1.10 (0.97, 1.24)	32	0.90 (0.65, 1.25)	41	1.17 (0.84, 1.63)	38	0.80 (0.65, 0.99)	31	0.96 (0.79, 1.17)
Unknown	14	0.79 (0.67, 0.92)	25	0.83 (0.59, 1.16)	36	1.04 (0.79, 1.36)	21	0.59 (0.40, 0.87)	13	0.48 (0.35, 0.65)
*p* for heterogeneity^b^		0.003		0.45		0.62		0.004		<0.0001

CI confidence interval, *NSCLC* non-small cell lung cancer, *SCLC* small cell lung cancer, *TRR* treatment rate ratio.

^a^ Proportion of patients recorded to receive treatment in each category

^b^ Performed jointly across all categories of each variable, using a post-estimation Wald test

### Variation by deprivation

Mostly, increasing deprivation quintile was associated with decreasing use of CT and CTRT (Tables 4 and 5). The steepest association was oesophageal cancer, as the TRR among patients in the most deprived quintile was 0.77 (95% CI 0.65, 0.91) for CT and 0.61 (95% CI 0.43, 0.87) for CTRT. Exceptions with no association were SCLC for CT, and stomach and pancreas for CTRT. There was no evidence of an association of deprivation with RT (*p* = 0.18; Table 2).

### Variation by Charlson comorbidity index

Increasing comorbidity index was associated with decreasing CT and CTRT rates (*p* < 0.0001; Table 2). The steepest association was for NSCLC and CTRT (the TRR among patients with a comorbidity index of 3+ was 0.35 (95% CI 0.27, 0.45)) (Table 4). The exceptions with no significant association were SCLC and CT (Table 5), and oesophagus, stomach and pancreas and CTRT (Table 4). For most sites, no association was found between comorbidity and RT, except for a positive association with SCLC (*p* = 0.02) of 1.80 (95% CI 1.17, 2.77) among patients with a comorbidity index of 3+ (Table 3).

### Association with performance status

Performance status (PS) was known for 52% of the cohort. Inclusion of PS made no substantive difference to the overall associations (Supplementary Table 6). However, there was a significant association with PS for all treatments (*p* < 0.0001). In stratified analysis (by broader PS groups), for example, no association was found between CTRT and comorbidity nor deprivation among patients with a PS of 0 (*p* = 0.62 and *p* = 0.47, respectively; Supplementary Table 7).

## DISCUSSION

Our population-based study of patients with stage IV lung, oesophageal, stomach and pancreatic cancer aimed to define uptake and variation in treatment use. The main finding was significant variation in the rates of CT and RT, for patients who receive one treatment alone or both within 6 months post diagnosis. As expected on clinical grounds, there was significant heterogeneity by cancer type, given the variable efficacy of different treatment modalities in different cancers. After accounting for this, variation was largely driven by age at cancer diagnosis, but non-trivial associations with comorbidity and deprivation were also observed, and additionally associations of sex with RT use. More variation in treatment appeared to align to where clinical guidelines are less clear. One example of this was the differential (by sex) use of RT among patients with non-squamous NSCLC tumours, where the indication for use of RT is less clear. Stratification by performance status demonstrated non-trivial adjustments to these associations suggesting that improved completeness of performance status may further explain variation in treatment, particularly as patients with unknown performance status appeared to have poor health status.

Over half of patients received neither CT nor RT within 6 months following diagnosis, although a significant fraction died quickly after diagnosis. Our study focused on RT and/or CT use, though these patients may have received other treatments, appropriately not been offered treatment on clinical grounds, received treatment in a private setting or chosen not to be treated. A small proportion of patients (estimates vary, but a recent study in London found 0.6% of breast cancer patients had been treated privately and did not appear in the cancer registration data^30^) are believed to be treated in the private setting, and as they are treated using standard clinical guidelines the incompleteness of this data is unlikely to impact the findings of this study. It is also possible that a limitation in data completeness has contributed to missing a fraction of the treatments.

### Variation in treatment rate by age at diagnosis and Charlson comorbidity index

We found large gradients in treatment use among patients with stage IV cancer for both CT and RT with increasing age, and non-trivial associations with increasing level of comorbidity. Such variations may be expected, as clinicians and patients must balance the benefit of palliative therapy against the toxicity of treatment.^17^ CT can be highly toxic in patients with multiple comorbidities, which is supported by our findings of reduced usage among those with more comorbidity. Palliative RT for advanced disease typically has a very low rate of side effects therefore would be expected to be very much less influenced by the presence of comorbidities. Our findings support this and are largely consistent with a study of non-surgically treated lung cancer patients.^1^ Despite clinical appropriateness in certain circumstances, under-treatment of elderly patients has been well-documented for a number of years;^18^ which may partly explain why in international comparisons of death rates the number of excess deaths in England are highest among older patients with cancer.^31–35^ Treatment rates of advanced disease do appear to be higher in the US for some cancer sites, although evidence is limited.^36^ In the United Kingdom, however, there has been shown to be an increasing use of chemotherapy for advanced NSCLC patients with low performance status;^15^ therefore, the discrepancy with the US study may be reducing. However, more treatment is not universally preferred, and there are circumstances where treatment may be more harmful, due to the health status of the patient and the potential impact of the side effects of treatment. Understanding the impact of variation on survival should provide further evidence for this.

The different treatment rates among older, compared to younger, patients were not explained by the presence of comorbidity, poor performance status or other demographic factors. Our study demonstrated positive associations between the likelihood of RT use and increasing age at diagnosis, and a negative association with the likelihood of CT and CTRT use. The association between increasing age at diagnosis and lower CT use concords with prior US literature.^7,37^ The association between increasing age and increasing RT, however, was in contrast to a recent Danish study,^1^ which is likely due to differences in stage distribution of the selected populations. In adjusted analysis, increasing RT use with increasing age was only observed among patients not treated by CT. This presumably reflects that RT is better tolerated among some elderly patients than CT.

### Variation in treatment rate by deprivation

A significant association with deprivation was demonstrated for patients treated with CT alone, and for patients treated with both CT and RT within 6 months but no association was found for RT. This was largely driven by patients with lung or oesophageal cancers. However, the association with CT alone was not apparent for SCLC patients, though we were unable to identify a clinical explanation for this. The deprivation deficit has been documented in previous studies of oesophagogastric cancer^38^ and lung cancer.^13,39,40^ Our work found the greatest association for CT. This confirms findings from a systematic review and meta-analysis of lung cancer treatment, which found a significant negative association between socioeconomic position and chemotherapy receipt, but no association with radiotherapy receipt.^41^ However, other published studies in England, which have accounted for travel time, have documented a more pronounced deprivation deficit in RT compared to CT treatment.^42,43^ This was largely attributed to travel times to RT centres. These local delivery issues may have reduced impact within a national study such as ours, and differences in the number of hospital attendances for palliative radiotherapy and chemotherapy delivery as compared to curative delivery may explain this discrepancy.

### Strengths and limitations

This study has population-based coverage, possible because of access to a wide range of routinely collected health care data in England. The availability of linked treatment datasets improved treatment ascertainment for multiple cancer types. There is now a high level of staging completeness which has allowed the reliable identification of a group of late stage cancer patients at a national level.^44^

Many patients with very advanced, stage IV cancers die very quickly following diagnosis and one strength of our methodology is that these patients, who would be very highly unlikely to receive treatment, have been censored. This allowed the estimates to be calculated using a patient’s time available for treatment therefore producing accurate estimates of the treatment rates and associations.

The main limitation of this study is that patient choice could not be accounted for. Inclusion of this information may attenuate or even help explain the associations with deprivation and age. Patient choice is influenced by many factors,^45^ and there is limited population-based data reporting patient choice. More deprived patients may be less inclined to accept the associated risks of treatment, the patient–clinician interaction may be impacted and understanding of the disease may differ,^46–48^ and there may be increased fatalism. Having adjusted for co-morbidities and, to a lesser extent, performance status, it seems unlikely that underlying ill-health is the only factor explaining the lower treatment rates in more socially deprived patients. Specifically, the differential impact of deprivation on the choice of active treatment was less apparent among those patients with a better performance status. However, we were not able to fully take account of frailty,^19^ smoking status or lung function, factors which are particularly relevant for lung cancer, though less so in the palliative setting. As always in population-based studies, there are also some limitations relating to data completeness, in particular incomplete data on performance status and the fact that our index of comorbidity is based only on in-patient hospital records. More complete performance status may have explained some of the remaining variation, particularly among the gastrointestinal cancers where completeness was the lowest. It is also worth noting that for oesophageal, pancreatic and stomach cancer, the staging data completeness increased markedly from 2013 to 2014, thus increasing the number of stage IV diagnoses in 2014 (Supplementary Table 8).

### Further research

This study has clearly demonstrated that unexplained variation in receipt of treatment exists among a cohort of stage IV cancer patients in England. However, it is not clear if this variation results in differences in expected survival for these patients. Statistical analyses may permit understanding of the excess mortality among these patients, and the attribution of any excess mortality to known patient and tumour characteristics, as in a study of short-term breast cancer mortality.^49^ This is vital further research among these patients diagnosed with stage IV disease. This information also requires further work to define why some patients, particularly the elderly, are not treated with either radiotherapy or chemotherapy, as barriers to patients receiving palliative treatment can often be overcome.^50^ This is an active area of research among patients with various cancer types in older patients.^51,52^

### Implications for policy and practice

The main finding is that older patients are, in general, receiving lower rates of active treatment for their cancers and that these low rates cannot be fully explained by co-morbidity. It is well known that there are higher excess death rates from a number of cancers in older patients in England than in some other countries. The explanation for this warrants further work and may well be largely related to patient choice influenced by cultural factors. Healthcare providers need to examine how they support older patients when making treatment decisions and policy makers need to improve the access of cancer teams to such tools as the Comprehensive Geriatric Assessment.^50–52^ Qualitative studies and ethnography are needed to understand the patient-clinician interaction, and its impact on patient choice of treatment. Data linkages to primary care information should be explored, to capture unmeasured morbidity which may explain the variation in treatment received among these patients.

## Conclusion

Contributing to a body of evidence documenting age and deprivation deficits in treatment for cancer patients, our study defines these deficits as being very evident among patients with advanced disease requiring essentially palliative treatments. Factors other than health status appear to be important in explaining this, as they remain after adjustment for recorded factors. Clinicians and service planners should consider these findings and future health policies should attempt to address them. Strategies should be developed to improve valid routine collection, processing and analysis of information on patient choice, to attempt to further understand this complex topic. Finally, our approach using routinely collected health data can potentially be used for routine surveillance across all cancer types, particularly for those cancers not currently covered by National Audit initiatives, thus to monitor trends in variation over time and improve outcomes for patients.

## Electronic supplementary material

Supplementary Table 1:

Supplementary Table 2:

Supplementary Table 3:

Supplementary Table 4:

Supplementary Table 5:

Supplementary Table 6:

Supplementary Table 7:

Supplementary Table 8:
